# The additional use of strain measurements for timing of treatment in severe aortic regurgitation

**DOI:** 10.1007/s10554-021-02515-6

**Published:** 2022-02-09

**Authors:** Christoph Sinning

**Affiliations:** 1 Department of Cardiology, University Heart & Vascular Center Hamburg, Martinistraße 52, 20251 Hamburg, Germany; 2 German Center for Cardiovascular Research (DZHK), Partner Site Hamburg/Kiel/Lübeck, Hamburg, Germany

**Keywords:** Aortic regurgitation, Echocardiography, Strain measurement, Outcome, Treatment

## Abstract

Assessment of severity and need for intervention in clinical practice often is in need for a thorough echocardiography regarding function of the ventricles and additional valvular dysfunction. Despite the indications recommended in the current guidelines, there is still need for further research to identify patients with a severe valvular dysfunction but a potential reversible status regarding the function of the ventricles. Strain imaging is suggested in the current literature to be an additional tool to identify ventricular dysfunction in the setting of preserved left ventricular ejection fraction.

The treatment of valvular heart disease is decided in the majority of cases by symptoms and presence of changes as measured with conventional 2-dimensional echocardiography [1, 2]. In patients with aortic regurgitation left ventricular ejection fraction (LVEF) has to be below ≤ 50% or left ventricular end systolic diameter > 50 mm whereby this measurement was suggested to be rather 25 mm/m^2^ corrected for body-surface-area (BSA) [1]. The adaptation in context with BSA might be important in patients with a small body size of < 1.68 m^2^ BSA or in patients with severe aortic regurgitation which have a large BSA but are not overweight [1]. Previous studies did show that the application of measurement of left ventricular global longitudinal strain (GLS) in asymptomatic patients with a preserved ejection fraction of > 50% had a worse outcome with an increased mortality if GLS was more positive than − 19% [3, 4]. As one of the most important features of aortic regurgitation is chronic volume load and dilatation of the left ventricle some researchers did suggest to index the strain values to the end diastolic volume of the left ventricle to account for the increased preload which might impact the measured strain values [3, 5].

In this background Grund and co-workers did investigate the additional use of layer-specific GLS values regarding the endocardium, the epicardium and the overall GLS value in patients with either preserved or decreased LVEF and chronic severe aortic regurgitation as the hypothesis was that more positive GLS values could be distributed differently between endo- and epicardium [6]. Thus, this might facilitate decision-making regarding surgery by describing an earlier damage of the myocardium as reflected only by conventional echocardiography or GLS in the current guidelines [1, 6]. The main outcome was persisting end diastolic volume of > 175 ml in the patients after a time period of 3 months. Although the conventional echocardiography variables with LVEF, end diastolic and end systolic volume were associated with persisting left ventricular dilation if pathologic values were described before surgery. Parameters which could identify patients without post-surgery dilation, besides normal values for the previous mentioned conventional echocardiography variables, were not described [6]. It is essential to note that even patients with normal LVEF did show a decline of LVEF after surgery and those individuals with impaired LVEF did show no improvement suggesting a persisting myocardial dysfunction of the left ventricle. In the work by Grund and colleagues the GLS and layer-specific GLS were all reduced in comparison to healthy controls and did show a further decline after surgery, thus these parameter did as well indicate the impaired function of the left ventricle being present at the time of surgery [6]. The concept of measurement of GLS was predictive of the defined outcome of persisting left ventricular dilation in this study [6] but as well regarding mortality being in line with previously described work [3, 4]. For the clinician an imaging parameter indicating a severe aortic regurgitation without persisting impairment of the LVEF following surgery would be ideal to augment the conventional echocardiography variables or the measurement of GLS [1, 3, 4]. Although layer-specific strain measurements are feasible and reproducible still there remains an inter vendor variation which renders a definition of a cut-off difficult in the clinical setting [7, 8]. The idea of the authors of the current study to show that patients with an impaired endocardial GLS without epicardial impairment might be individuals without persisting dilation of the left ventricle is of merit as other authors could describe impaired GLS only in patients with severe aortic regurgitation [9]. Thus, it might be an option to measure layer-specific strain to detect presence of beginning decline of LVEF mirrored by impaired endocardial GLS in the presence of normal epicardial GLS. However the authors of the current study could not report these findings and the outcome of this cohort of patients still underlines the need for additional imaging in patients with the presence of severe valvular heart disease to identify types of valvular heart disease were intervention is indicated. Although the study could not answer this question, important findings in the context of severe aortic regurgitation were that layer-specific GLS is decreased and as well is associated with a persisting decrease in LVEF which was not described in a previous study so far [6]. Further, these measurements can be integrated into the preoperative echocardiography allowing for an additional parameter during the follow-up of the patient. An important point described in the study of Grund and co-workers [6] was as well that measurement of layer-specific strain and measurements of strain indexed to end diastolic volume were not superior to measurement of GLS without adjustments to detect the defined outcome of the study [6]. This underlines previous results that impairment of GLS is an additional parameter indicating an increased mortality during follow-up [3, 4]. As of the current data measurement of GLS is advisable were possible to have additional information to identify patients with a potential high risk for a poor outcome being in need for a closer follow-up. However, there is no data which favour the measurement of layer-specific GLS outside scientific studies as there is no potential additional information currently derived from the measurement in comparison to GLS.

In summary the study by Grund and colleagues is an important work advocating the additional measurement of GLS in the setting of severe aortic regurgitation and adds the additional hypothesis of layer-specific GLS to detect early stages of ventricular dysfunction but this suggestion is in need of future additional research.
